# Supplementary material for: Prognostic Factors of the Inability to Bear Self-Weight at Discharge in Patients with Fragility Femoral Neck Fracture: A 5-Year Retrospective Cohort Study in Thailand
Source: Int J Environ Res Public Health. 2022 Mar 28;19(7):3992. doi: 10.3390/ijerph19073992 (PMC8997639; doi:10.3390/ijerph19073992)
Supplement: Supplementary file 1 [file ijerph-19-03992-s001.zip › ijerph-1587156-supplementary.pdf]

Supplementary Table S1: Power back calculation based on the current sample size and the required number of samples needed to reach statistical significance for each prognostic variable.

	Unable to bear self-weight at discharge (n=55) n (%)	Able to bear self-weight at discharge (n=214) n (%)	p-value	Power	Required Sample size*	Adequate sample size
**Sex**						
- Male	19 (34.55)	57 (26.64)	0.245	0.266	1,267	No
- Female	36 (65.45)	157 (73.36)				
**Age ≥ 80 years**	27 (49.09)	100 (46.73)	0.764	0.069	17,000	No
**BMI ≥ 25 kg/m²**	8 (14.81)	41 (19.34)	0.557	0.128	2,668	No
**Underlying diseases**						
- ESRD	11 (20.00)	13 (6.07)	***0.003***	0.844	195	Yes
- Cirrhosis	3 (5.45)	0 (0)	***0.008***	0.710	263	Yes
- Cerebrovascular diseases	10 (18.18)	17 (7.94)	***0.040***	0.616	377	No
- Psychiatric disorders/Drug abuse	6 (10.91)	5 (2.34)	***0.011***	0.718	269	Yes
- Parkinson disease	2 (3.64)	6 (2.80)	0.668	0.078	16,400	No
- Diabetes mellitus	12 (21.82)	48 (22.43)	1.000	0.058	176,000	No
- Heart diseases	12 (21.82)	36 (16.82)	0.430	0.178	2,319	No
- COPD/Asthma	5 (9.09)	15 (7.01)	0.571	0.094	6,345	No
- Eye diseases	4 (7.27)	16 (7.48)	1.000	0.078	590,000	No
- Cancer	3 (5.45)	16 (7.48)	0.773	0.061	5,719	No
- Dementia	5 (9.09)	19 (8.88)	1.000	0.078	703,000	No
**Pre-fracture ambulation status**						
- Independent ambulation	29 (52.73)	166 (77.57)	***<0.001***	0.958	130	Yes
- Ambulation with gait aids	22 (40.00)	48 (22.43)		0.778	251	Yes
- Ambulation in wheelchair	2 (3.64)	0 (0)		0.557	412	No
- Non-ambulatory status	2 (3.64)	0 (0)		0.557	412	No
**Hypoalbuminemia** (<3.5 g/dl)	23 (50.00)	48 (26.97)	***0.004***	0.919	161	Yes
**Associated fracture**	10 (18.18)	2 (0.93)	***<0.001***	0.982	83	Yes
**Second hip fracture**	6 (10.91)	20 (9.35)	0.798	0.068	14,000	No
**Surgical technique**						
- Arthroplasty	41 (74.55)	165 (77.10)	0.722	0.081	10,600	No
- Fixation	14 (25.45)	49 (22.90)		0.081		
**Time delayed from admission to surgery >48 hours**	51 (92.73)	174 (81.31)	0.042	0.597	344	No
**Anesthesia time (hours)** [a]	2.17 (1.92, 2.50)	2.00 (1.75, 2.25)	0.010‡	0.588	296	No
**Intra-operative blood loss (ml)** [a]	100 (50, 200)	100 (90, 200)	0.712‡	0.368	488	No
**Post-operative ICU admission or ventilator used**	10 (18.18)	4 (1.87)	<0.001	0.966	101	Yes
**Major post-operative complications**	9 (16.36)	5 (2.34)	<0.001	0.926	132	Yes
**Other operation in admission**	5 (9.09)	4 (1.87)	0.020	0.657	317	No
**Post-operative sedative drug used**	28 (50.91)	58 (27.10)	0.001	0.933	151	Yes
**Post-operative blood transfusion**	18 (32.73)	50 (23.36)	0.166	0.355	851	No
**Urinary catheter used at post-operative day 2**	22 (40.00)	38 (17.76)	0.001	0.935	146	Yes
**Moderate to severe pain score at rehabilitation day (PS=4-10)**	7 (14.29)	30 (14.56)	1.000	0.068	645,000	No
**Pressure sore**	3 (5.45)	1 (0.47)	0.028	0.605	343	No

‡ Mann-Whitney U test, [a] Median (IQR), * Required sample size for event and non-event to achieve 80% power
